# Quantifying the Relationship Between Key Potential Environmental and Nutritional Health Benefits of Plant-Based Meat Alternatives and Their Price Premiums Compared to Beef

**DOI:** 10.3390/foods15111949

**Published:** 2026-06-01

**Authors:** Mauricio R. Bellon, Kathleen Merrigan, Christopher Wharton

**Affiliations:** 1 Swette Center for Sustainable Food Systems, Arizona State University, 777 East University Dr, Tempe, AZ 85281, USA; kathleen.merrigan@asu.edu; 2 College of Health Solutions, Arizona State University, 7231 E Sonoran Arroyo Mall, Mesa, AZ 85212, USA; cwharton@asu.edu

**Keywords:** plant-based meat alternatives, beef burgers, negative externalities, monetization, true cost accounting

## Abstract

Beef production and consumption generate significant environmental and health costs for society, negative externalities that are not generally reflected in retail prices. Plant-based meat alternatives (PBMAs) are products designed to emulate conventional meat by using plant-based ingredients that are purported to produce significantly fewer negative externalities than beef but are often substantially more expensive. Are the premiums paid by consumers for PBMAs worth the potential environmental and nutritional benefits received by society from choosing them instead of beef? To address this question, we analyze the impacts of two well-known PBMAs in the USA, Beyond Burger^®^ and Impossible Burger^®^, compared to those of beef on global warming potential, water consumption, dietary risks, and the market retail prices of each product. Results show that the public benefits of Beyond Burger^®^ and Impossible Burger^®^ are equivalent to USD 2.39 and 2.31, while additional private costs are USD 0.81 and 1.08 per patty, indicating ratios of 2.96 and 2.13, respectively, between the public benefits and the premiums paid by consumers. These results, while conditional on these specific products, the datasets available, and assumptions of the methods used, suggest that some level of public support for PBMAs may be justified.

## 1. Introduction

Beef production and consumption generate significant environmental and health costs for society [1,2,3,4,5]. Most of these costs are not reflected in the market price of beef; as such, they constitute externalities [5,6,7]. An externality is “a positive or negative consequence of an economic activity or transaction that affects other parties without being reflected in the price of the goods or services transacted” [8] (p. 29). Ignoring these costs in market transactions is likely to lead to suboptimal decision-making for individuals and society [6,7,8,9]. For example, beef production is a leading emitter of methane, a very powerful greenhouse gas and major contributor to climate change; however, this environmental cost is not included in the price of beef, leading to significant price distortions and welfare losses [6,10]. Incorporating these external costs in individual and public decision-making is fundamental as we aim to move to more sustainable food systems [11,12]. Furthermore, given the global pattern of increasing beef production and consumption, these external costs potentially outweigh the high quality of protein generally prized in beef products [1].

Over the last 20 years, there has been a growing popularity of plant-based meat alternatives (referred here as PBMAs in plural, PBMA in singular) [4,13,14,15,16,17]. PBMAs are products designed to emulate conventional meat with respect to appearance, taste, smell, functionality and cooking experience by using plant-based ingredients such as soy, mung, and fava beans, peas, wheat, mushrooms, and rice [14,18]. Although there are many reasons consumers choose these products, concerns about the environment, health, and animal welfare are important considerations [4,17,19]. In particular, PBMAs are considered to produce significantly fewer negative externalities than beef, such as lower greenhouse emissions, water consumption, and land use [4,13,16,20,21,22,23,24].

The market for meat substitutes has expanded, with global sales from PBMAs likely to exceed USD 33 billion by 2027 [13]. Growth is likely to continue due to concerns about the environmental impacts of meat and a desire for healthy diets [25]. Even so, and despite the magnitude of these sales, this market’s growth may be slowing due to some consumers’ somewhat inelastic preference for traditional meat products, as well as perceptions that meat alternatives are overly processed—which could convey a sense of unhealthiness—as well as concerns about flavor and texture, convenience, and price [13,26]. Also, given that PBMAs are considered ultra-processed foods, segments of consumers harbor concerns about potential harmful effects associated with these types of foods [27], even though there is evidence that PBMAs are an exception in the ultra-processed food category, given their healthier nutrient profile compared to the foods they aim to replace [28]. PBMAs can also be substantially more expensive than meat [4,14,16,20], an important factor limiting their wider consumption [20,29,30]. The difference in price between a PBMA and the type of meat it aims to replace is commonly referred to as the price premium [20,31].

The objective of this paper is to quantify the relationship between the additional private costs incurred by consumers of PBMAs, in terms of the added retail price they pay, compared to the public benefits that society receives from their decision to purchase these products instead of beef (here on, referred to as the benefit-to-premium relationship). These public benefits can be conceptualized as the quantity of negative externalities avoided by consuming PBMAs instead of beef. Specifically, we address the question: are the price premiums paid by consumers for PBMAs worth the additional environmental and nutritional benefits received by society from choosing them instead of beef? Addressing this question is important for understanding how to move to more sustainable food systems that balance the consumption of plant and animal derived foods, but doing so with an understanding of the costs borne by individuals, society, and the planet [4,10,32]. While there are multiple studies that have examined the differences in environmental costs and nutritional properties of PBMAs versus beef (e.g., [4,22,23,24]), as well as a few studies on the true costs of specific food products [33,34,35], to our knowledge the broad issue of quantifying the relationship between price premiums and externalities of different food products has yet to be addressed. Measuring the benefit-to-premium relationship of PBMAs relative to beef could provide important insights into whether and how much public support for innovation and investment in PBMAs there could or should be. This is particularly important given the likely need to reduce PBMAs’ market price should there be interest in enabling their wider consumption. The importance of this issue is underlined by the recent report by the EAT-Lancet Commission on healthy, sustainable, and just food systems that recognized the potential contribution of PBMAs to the transition to more sustainable food systems but noted the challenge of their higher price compared to the products they replace [36].

## 2. Conceptual Approach, Methods, and Data

To address this question, we quantify the relationship between the additional public benefits and private costs of a PBMA versus beef as a benefit-to-premium ratio (Equation (1)):

(1) R=(Epbma−Ebeef)/(Ppbma−Pbeef)

where

*R* is the benefit-to-premium ratio;
*Epbma* is the external cost of the PBMA;
*Ebeef* is the external cost of beef;
*Ppbma* is the retail price of the PBMA;
*Pbeef* is the retail price of beef.

The benefit-to-premium ratio *R* quantifies the magnitude of the public benefits associated with a PBMA compared to beef, relative to the differences in retail prices between both, i.e., the premium consumers pay for a PBMA compared to beef. Both external costs have to be monetized first to be able to calculate the ratio *R*. Furthermore, assuming that in fact meat generates higher external costs than the PBMA, the nominator in Equation (1) should be negative, indicating a benefit due to the negative externalities avoided by consuming the PBMA instead of beef. Therefore, although the benefit-to-premium ratio *R* should be negative, for simplicity we report its absolute value.

To operationalize Equation (1), we use a true cost accounting (TCA) methodology from previously published work in which five plant-based milk alternative beverages were compared to dairy milk [34]. TCA is an economic assessment aimed at making visible, measuring, and if possible monetizing critical positive and negative externalities in food systems [11,12,37]. Externalities are assessed based on the reliance on, and effects of, an agri-food value chain in four capitals: natural, human, social, and produced [8]. Natural capital refers to “the limited stocks of physical and biological resources found on earth, and of the limited capacity of ecosystems to provide ecosystem services” [8] (p. viii), and is commonly operationalized by quantifying environmental costs derived from Life Cycle Assessments (LCAs) [34]. Human capital refers to the knowledge, skills, competencies, and attributes embodied in individuals that facilitate the creation of personal, social, and economic well-being [8], of which diets and nutrition are crucial for human health and thus a central component of human well-being [34]. Produced capital refers to “all manufactured capital, such as buildings, machinery, physical infrastructure (roads, water systems), as well as all financial capital and intellectual capital (technology, software, patents, brands, etc.)” [8] (p. viii), and is relatively straightforward to measure since market prices are associated with it [34]. Social capital “encompasses networks, institutions, shared norms, values and understanding that facilitates cooperation among groups” [8] (p. viii) and is probably the most difficult to measure at the scale of a specific product [38].

Although beef and PBMAs generate numerous different types of externalities, given the nature of our question, we only focus on externalities where reliable data are available and can be monetized. The point of monetization in TCA is not to commoditize or privatize nature or other nonmarket aspects of food systems, but to provide more transparency about the tradeoffs involved to facilitate more sustainable decisions that can concretely be “valued” by decision-makers [12]. Monetization allows the aggregation of very different impacts and a more transparent comparison among them [38].

The specific focus of this paper is on burger patties, which are the leading product in the market for plant-based beef alternatives and capture the largest estimated global market value among PBMAs ($2.1 billion in 2022), particularly in the USA [14]. To address our question, we present two case studies based on the published data from two of the most popular brands of PBMAs in the USA [39]: Beyond Burger^®^ 3.0 [40] and Impossible Burger^®^ [41]. Beyond Burger^®^ is a pea and rice protein-based patty designed to look, cook, and taste like a beef burger [40]. Impossible Burger^®^ is a soy and potato protein-based patty that incorporates heme that provides a meaty flavor and color. Heme is obtained through the fermentation of naturally occurring leghemglobin expressed by a genetically modified yeast [41]. These are two different independent studies with different structures and assumptions. Thus, they are not strictly comparable. Here, we compare separately the performance of each PBMA to a beef patty regarding global warming potential (GWP) and water consumption, representing natural capital; dietary risks, representing human capital; and the market retail prices of these products, representing produced capital. Unlike previous studies [38,42], we do not analyze indicators of social capital due to data limitations. Therefore, our study is not a comprehensive true-cost assessment since only selected components of the natural and human capitals are analyzed. Furthermore, while cross-product comparisons are not appropriate, comparisons of the PBMA to the respective beef patty within each study are valid. We present both cases side-by-side to illustrate our approach under different circumstances.

### 2.1. Environmental Impacts

The environmental data are based on the published results of LCAs of Beyond Burger^®^ [40] and Impossible Burger^®^ [41]. In each of the LCAs, the environmental impacts of the PBMA were compared to the impacts of beef produced in the USA. Table 1 presents key parameters from each LCA. The functional units for the PBMAs and their beef comparison are different between studies in terms of the final product and units used. The data for the beef comparisons have been produced by the same group of experts [43,44,45,46]. The system boundaries are similar. The allocation methods are slightly different. The Life Cycle Assessment Impact (LCIA) methods are different, and while both studies report on impacts on climate change, land use, and water use, the resulting environmental impacts are not strictly comparable. Given the differences between both studies, comparisons are made within each study and not across them; thus, the analyses are presented separately. However, all results are reported normalized to the same unit, a 4 oz patty (0.113 kg), for ease of presentation. Since the focus of the paper is on estimating the monetized value of externalities, we report only on the impacts on GWP and water use, which are reported in both studies and for which monetization factors are readily available.

### 2.2. Nutritional Health Impacts

We use the Health Nutritional Index (HENI) developed by Stylianou et al. [48] to assess and compare the nutritional health impacts of the PBMAs to a beef patty (80% lean/20% fat). Both studies use this type of beef patty for a nutritional comparison of their products. It should be noted that “the ratio of lean meat to fat does not influence the LCA results” [40]. The HENI is based on “the marginal health burden associated with 15 dietary risks, defined as dietary risk factors (DRFs) and expressed in disability-adjusted life years (DALYs) per g intake of risk component” (p. 616). DRFs were calculated for the diets of US adults (aged ≥25 years) [48]. Because DALYs can increase or decrease, the HENI covers both health benefits as well as negative health impacts. We calculated the dietary risks associated with each of the PBMAs and the beef patty based on their content of the nutrients and associated dietary risk factors that are relevant among the 15 dietary risks of the index. These nutrients include polyunsaturated fatty acids (PUFAs), trans fatty acids (TFAs), calcium, sodium, and fiber. In addition, Stylianou et al. report a specific DRF for red meat associated with its contribution to the risk of colorectal cancer and Type 2 Diabetes mellitus (Supplementary Information of Stylianou et al. [48]), which we included in the calculations for the beef patty (a detailed explanation and the relevant data are presented in Supplementary Materials Text S1). We calculated the total dietary risk (TDR) of each product as follows (Equation (2)):

(2) $$TDRj=\sum_{i}DRFi\times Nij$$

where

*TDRi* is the total dietary risk of product *j* in µDALY/gram.
*DRFi* is the dietary risk factor of nutrient *i* in µDALY/gram.
*Nij* is the content of nutrient *i* in product *j* in grams.

The nutrient contents of the relevant nutrients for the PBMAs were obtained from Harnack et al. [49] and are based on nutrient composition information available in the 2020 version of the University of Minnesota Nutrition Coordinating Center Food and Nutrient Database. They report on the nutrient contents of several PBMA brands, including those analyzed here. In this database, the nutrient content of each PBMA has been modeled into a standardized 3 oz portion from the nutrient content of a 4 oz uncooked portion obtained from each product nutrition fact label, adjusted for shrinkage due to cooking [49,50]. The nutrient content for the beef was obtained from direct measurements from the U.S. Department of Agriculture FoodDataCentral for broiled ground beef 80% lean/20% fat patty for a 3 oz portion [51]. Cooked beef tends to shrink by about a fourth of the uncooked weight [52]. The environmental coefficients are based on a 4 oz uncooked portion, while the HENI calculations are based on a 3 oz cooked portion. We considered them equivalent since the 3 oz cooked portion is derived from a 4 oz uncooked portion for the PBMAs and the beef patty. Therefore, when addressing environmental results, we report them in terms of a 4 oz uncooked patty, while when addressing nutritional results and when both results are combined, we report them in terms of a 3 oz cooked patty. Because nutrient content of beef is the same for both studies, we do not carry out distinct comparisons for each PBMAs as we have done for the environmental impacts.

An important difference between beef and the PBMAs is that only beef contains TFAs. However, these are naturally occurring TFAs which some research has suggested might not be as harmful as those derived from industrial processes [53,54]. Importantly, evidence remains mixed, and they are likely to have, if any impact at all, a negative one [55]. Even so, given that TFAs contribute 25% to the TDR of a beef patty, we also examine the sensitivity of our results considering TFAs as having a neutral health effect.

### 2.3. Monetization

There are different monetization factors available, based on different valuation approaches [56,57]. Here we use monetization factors that are well documented [57,58,59] and used in other TCA studies [33,34,35,60]. To monetize the GWP reported by both studies, we use an estimate of the social cost of greenhouse gases reported by the U.S. Environmental Protection Agency (EPA) [59] of USD_2020_ 120/ton of CO_2_-eq for emissions that took place in 2020. For water consumption, we use the monetization factor of scarce blue water (surface and ground water) use reported by the True Price Foundation [61] of USD_2022_ 1.56/m^3^. The monetization factor of USD_2022_ 125,000 for a DALY was obtained from True Price Foundation [61]. For compatibility with the data on retail market prices of the PBMAs (see below), these factors were deflated to USD of April of 2025 using the Consumer Price Index for All Urban Consumers (CPI-U), not seasonally adjusted [62], resulting in the following monetization factors: USD_2025_ 149,000/ton of CO_2_-eq for GWP, USD_2025_ 1.17/m^3^ for water consumption, and USD_2025_ 137,019.27/DALY for the nutritional health impacts.

Retail market prices for both PBMAs and for a beef patty (80% lean/20% fat) were obtained from the website of a major US retailer (Walmart.com) for online purchases in April 2025. Walmart is the largest grocery retailer in the country with a strong e-commerce presence [63]. The retail price data used here do not consider its variability through time and location, and this is an important limitation of this study. Obtaining this type of disaggregated data for specific PBMAs can be expensive and difficult since it is usually proprietary (e.g., [64]). However, given that for our analysis the relevant relationship is the difference between the retail prices of a PBMA and its beef alternative, we carried out a sensitivity analysis by modifying this difference, reflecting potential variation in the underlying prices (see below).

### 2.4. Uncertainty

To address uncertainty related to the monetized external costs estimated here we use the 95% confidence intervals (CIs) reported in each of the studies for the PBMAs and their beef comparisons. The Beyond Burger^®^ LCA provided 95% CI for the environmental impacts of the PBMA based on Monte Carlo simulations using 500 iterations, but not equivalent data for the beef comparison [40]. The Impossible Burger^®^ LCA provided 95% CI for the environmental impacts based on Monte Carlo simulations using 1000 iterations for both the PBMA and the beef comparison [41]. Stylianou et al. [48] provide 95% CI for each nutrient dietary risk factor, which we use to calculate 95% CI for the HENI associated with the PBMAs and the beef patty (Text S2 in Supplementary Materials describes the specific methodology employed for these calculations and presents the detailed data used). These data were utilized to calculate 95% CI for the monetized external costs and true costs. As indicated above, the environmental uncertainty for Beyond Burger^®^ is incomplete, since the respective LCA does not provide CI for the beef comparison. Therefore, the uncertainty treatment is asymmetric across products.

To address uncertainty regarding monetization factors, we carried out sensitivity analyses for our results using other monetization factors. For GWP, we used a widely cited earlier value reported by the EPA of USD_2020_ 51/ton of CO_2_-eq [58] as a lower bound, and another from the True Price Foundation [61] of USD_2022_ 236/ton of CO_2_-eq as an upper bound. For the monetization factor for the DALY, we used a value of USD_2013_ 23,000/DALY from the LIME 3 LCIA method [56] as a lower bound. We estimated a baseline and three scenarios: (1) “maximum” using the highest social cost of carbon and monetization factor for the DALY, (2) “widest spread” combining the highest social cost of carbon with the lowest value for the DALY, and (3) “minimum” with the lowest values for the social cost of carbon and the DALY. Each scenario was also estimated for varying price differences between the retail prices of a PBMA and its beef alternative by changing these differences from −20% to 20%. As indicated above, we also carried out these analyses for the assumption that TFAs have a neutral health effect. For the sensitivity analyses, these alternative monetization factors were deflated to USD of April of 2025. We were not able to identify alternative monetization factors for water consumption, so variation in this impact was not addressed in our analysis, which is another limitation of our study.

## 3. Results

### 3.1. Environmental Impacts

Table 2 presents the environmental impact coefficients and their monetization for Beyond Burger^®^ and Impossible Burger^®^ and their corresponding beef comparisons. As noted in the original reports, the GWP and the water consumption of the PBMAs are substantially lower than those of the beef patty. The PBMAs are about 10 and 11.5% of the GWP and 2.9 and 12.5% of the water consumption of those of beef in the respective comparisons. The studies reported dissimilar results for beef regarding environmental coefficients. This disparity confirms the appropriateness of carrying a separate analysis for each PBMA. The monetized value of the environmental impacts exhibits much larger impacts for beef than for the PBMAs in both studies. It shows that the contribution of GWP is substantially higher than that of water consumption to the external costs for each of the PBMAs and their respective beef comparison. The external costs of Beyond Burger^®^ are only 7.4% of its beef comparison, while Impossible Burger^®^ costs are 12.5% of its beef comparison. Although both studies are independent, the PBMAs show substantial savings in terms of environmental external costs compared to a beef patty in both cases.

### 3.2. Nutritional Health Impacts

Table 3 shows the nutrient content of both PBMAs and of beef used to calculate the HENI. Both PBMAs have substantially higher content of PUFA than beef, as well as dietary fiber, which beef does not contain. Calcium content is substantially higher for Impossible Burger^®^ compared to beef and Beyond Burger^®^, which has the lowest content. The beef patty is the only product with TFAs, but it also contained a lower content of sodium than both PBMAs, with Impossible Burger^®^ having the highest content overall.

Table 4 shows the mean dietary risks associated with the nutrient content of each product (Table S5 in Supplementary Materials present also the lower and upper bounds of a 95% CI for each nutrient and TDR by product). A negative sign indicates a benefit (a health burden avoided), while a positive sign indicates a cost (a health burden incurred). The TDR of beef is substantially higher compared to both PBMAs, mostly due to dietary risks associated with red meat and TFAs, which together account for 96% of the TDR. The lowest calculated was for Impossible Burger^®^, which is negative, indicating a health benefit, mostly related to the high calcium content compared to the other two products. The monetized TDR values for a beef patty and Beyond Burger^®^ show health costs of USD 1.63 and 16 cents respectively. The large contribution of the dietary risks associated with red meat and TFA show how beef is structurally different from the PBMAs in the nutritional index.

### 3.3. True Cost

Bringing both environmental and nutritional health external costs together shows that Beyond Burger^®^ generates only 9% of the external costs generated by a beef patty. For Impossible Burger^®^ the negative TDR implies a net nutritional benefit by compensating for the environmental costs when both are monetized and added together (Table 5). It should be noted that nutritional health costs are higher than the environmental costs for both PBMAs and their beef comparisons. Adding the market retail price of these products to their external costs to obtain their true cost shows that while the retail price of a beef patty is 41% and 48.2% cheaper than Beyond Burger^®^ and Impossible Burger^®^, respectively, the true cost of a beef patty is 71.7% and 54.6% higher than Beyond Burger^®^ and Impossible Burger^®^, correspondingly, though these results depend on the specific conditions under which these comparisons were made.

Table 6 presents the results of calculating Equation (1). It shows that while Beyond Burger^®^ costs an additional USD 0.81 compared to a beef patty, it generates external benefits equivalent to USD 2.39, showing a ratio of almost three times in additional public benefits compared to its additional private cost. Impossible Burger^®^ costs an additional USD 1.08, and it generates external benefits equivalent to USD 2.31, resulting in a ratio of slightly more than two times the additional public benefits compared to its additional private cost. These results indicate that in the two independent case studies we analyzed, the savings on external costs by consuming PBMAs instead of a beef patty are 196% higher for Beyond Burger^®^ and 113% higher for Impossible Burger^®^ than the additional price paid for them. Clearly, for both PBMAs, the benefits that they generate more than compensate for the price premiums they command.

Assume a neutral effect of TFAs reduces the external benefits of the PBMAs compared to beef from USD 2.39 to 1.99 in the case Beyond Burger^®^, with an associated reduction in the benefit-to-premium ratio from 2.96 to 2.47. In the case of Impossible Burger^®^, external benefits diminish from USD 2.31 to 1.91, and the benefit-to-premium ratio from 2.13 to 1.76. Therefore, even a 25% reduction in the value of nutritional health externalities reduces the ratio by only ~17% for both PBMAs (Table 7, Panel A). Assume a neutral effect for calcium reduces the external benefits of Beyond Burger^®^ minimally (USD 0.02), and of Impossible Burger^®^ very slightly (about USD 0.08), translating into a relatively small change in the benefit-to-premium ratio of this product from 2.13 to 2.06 (Table 7, Panel A). Under both assumptions, the benefit-to-premium ratios of both PBMAs are substantially greater than one, indicating larger external benefits relative to private costs.

In addition, results are robust under different assumptions about the social cost of carbon and monetization factors for the DALY, as well as for varying differences in retail prices between the PBMA and their beef comparison (Table 8). In the case of the retail prices observed, in all scenarios the benefit-premium ratio is above one, except for the minimum scenario for Impossible Burger^®^. In the cases of varying the difference in retail prices, only with a 20% increase in this difference does the benefit-premium ratio fall below one for both PBMAs. As such, in nearly all calculated scenarios across product studies the benefit–premium ratio is above one, indicating that the public benefits exceed the private costs associated with these two brands of PBMA.

## 4. Discussion

The results presented here provide an answer to the question posed in this paper. The premium paid for the PBMAs compared to a beef patty is worth the higher cost, by substantial margins, relative to the monetized value of the external environmental and nutritional health costs saved. This is the case even under more conservative scenarios accounting for unsettled nutrition-related factors such as naturally occurring TFAs. Identifying and quantifying these relationships are key contributions of our paper. Our approach synthesizes multiple secondary data and methods and developed metrics for addressing the balance between the private costs and the public benefits of market choices. To the extent of our knowledge, no prior study has addressed these issues, particularly in the context of PBMAs and beef. Even so, despite focusing on market leading PBMA brands, our results only refer to two specific PBMAs, and there are many other types and brands available for purchase [29]. Therefore, specific results may vary according to the products considered for evaluation and technologies that underpin them. This, along with other limitations noted throughout, such as the limited scope of price data used or the use of only one monetization factor for water, help to frame the approach we adopted as an in-depth place to start, but one that can evolve over time.

Other key considerations are important to highlight in order to further this work for these and other products in the future. While the higher environmental costs of beef compared to PBMAs are consistent with other studies [6,21,22], the most significant portion of the public benefits of PBMAs are associated with savings in nutritional health external costs compared to beef. These costs in beef are associated with the dietary risk factors of red meat and TFAs in the HENI, which are absent from the PBMAs considered here. Furthermore, the calcium content of Impossible Burger^®^ (10% of the reference Daily Value [DV]) played a beneficial role compared to beef and Beyond Burger^®^ due to their low content (2% and 1% of the reference DV respectively) (Table S1) [46]. It is difficult to know how important a role calcium could functionally play due to variability in bioavailability, which might be lower compared to other foods due to compounds in these products’ food matrices that might inhibit absorption or utilization (e.g., antinutrients) [27]. However, even assuming non-contribution of the calcium content in the HENI, results for Impossible Burger^®^ do not change significantly. The same was true when we assumed a neutral effect for TFAs in the HENI of beef.

Other relevant micro- and macro-nutrient as well as bioavailability differences exist between PBMAs and beef, for example, among iron, zinc, niacin, riboflavin, and vitamins B6 and B12, as well as in protein quality. These are not considered in the HENI (discussed in more detail in Supplementary Material Text S3). Further, the health implications of nutrient differences between PBMAs and meat are contextual and complex with specific implications depending on overall dietary patterns and characteristics of a population [27]. As such, the HENI method captures only a subset of these differences. As noted in previous published work, the HENI is “based on a narrow set of nutrients and assumes a linear association to health impacts, ignoring interactions among nutrients, and potential nonlinear relationships between a nutrient and a health effect, overlooking as well qualitative differences within a nutrient and assuming neutral health effects of all other nutrients not included in the calculation of TDR” [32] (p. 14). Therefore, we emphasize that our results are based on this index, and thus subject to its assumptions and limitations. The HENI only provides a partial approximation to the comparative nutritional health impacts of different foods that may not necessarily map onto real nutritional outcomes. Even so, it is likely to capture the most important impacts on health and remains useful as a first approximation for quantifying and monetizing these impacts.

Other limitations include the limited scope of the comparisons, and not addressing the role of government subsidies received by the products analyzed here. While we considered a key set of negative externalities, other externalities of potential importance were not included due to data limitations, such as impacts on land use, biodiversity, toxicity, eutrophication, etc. From a TCA perspective, we use a specific set of indicators for the natural, human, and produced capitals, while social capital was not addressed in our assessment. We also have not included possible other benefits that beef production may generate, such as its contribution to livelihoods, employment, and the use of marginal lands unsuitable for human agricultural uses other than as rangelands [3,15,42]. Therefore, our results only present a partial perspective on these issues. Finally, although it has been argued that that the animal farming sector in general, and beef production in particular, receive much greater government support than the plant-based alternative food sector in the USA and the European Union [65], accounting for government support in the calculations of the true costs of beef and PBMAs is complex and merits specific research that is beyond the scope of this study.

## 5. Policy Implications

A particular challenge for the wider consumption of PBMAs is that research consistently shows that consumers in the USA prefer beef over PBMAs, with only a relatively small share choosing the latter and willing to pay the premiums that these products command [13,14,19,20,66]. Other research has shown that PBMAs are a complement [14,64,67,68] or at most a relatively weak substitute for beef [31]. Furthermore, economic modeling suggests that for every 10% reduction in the price of PBMAs, there may be a reduction in global emissions equivalent to 0.34% of US greenhouse emissions from beef production, thus having a minor impact on emissions [69]. The results presented here suggest that the potential of PBMAs to reduce environmental and health externalities may be important. However, a recent study concluded that reducing the price of PBMAs beyond price parity with beef to increase their affordability might be key for supporting an enhanced shift to plant-based diets and therefore maximizing the potential for environmental and other public benefits from the consumption of PBMAs [20].

The public benefits of PBMAs can only contribute on a large scale if consumers consider them an acceptable substitution for beef [64]. However, their current price premiums seem to be a crucial deterrent for accomplishing this goal. The plant-based foods industry recognizes that the significant price premiums of PBMAs are a barrier to their continuing and wider purchase, noting that reducing the price gap between PBMAs and their animal counterparts will require public and private investments to scale up production and reduce their overall cost [29,70]. Although our results are limited and illustrative, they suggest that there may be large public benefits associated with a wider consumption of PBMAs should externalities become more apparent to consumers, particularly since as their price drops the benefit-to-premium ratio increases further. This in turn may justify increasing public support for innovation and investment in them, particularly to reduce their market price. This is an issue that merits further research.

## 6. Conclusions

Although the study presented here is limited to two specific PBMAs, it has illustrated that the public benefits of PBMAs in the USA could be substantial compared to the additional private cost incurred by consumers from their purchase. These results point to a broader challenge for the PBMA industry. While the public benefits of replacing animal meat with PBMAs may be high, the large premiums they command are a crucial barrier to their wider consumption, and thus for generating larger societal benefits. Therefore, reducing these premiums is key to unlocking these benefits at a larger scale. While PBMAs may never replace beef completely (and may not need to), they can contribute to the reduction in its consumption and associated negative externalities with further private investment and potentially public sector support. Our results suggest that public investment and support may be justified given the potential public benefits that could be generated for society.

## Figures and Tables

**Table 1:** Comparison of key parameters of LCA studies.

Parameters	Beyond Burger^®^	Beef Comparison	Impossible Burger^®^	Beef Comparison
Functional Unit	4 oz—patty (0.113 kg) ^1^	4 oz—beef patty (0.113 kg)	1 kg ^2^	1 kg of conventional ground beef
Source of data for the beef comparison		Putman, Rotz, and Thoma [44].		Assem-Hiablie et al. [43]; Rotz et al. [45,46].
System boundary	Cradle-to-distribution	Cradle-to-distribution	Cradle-to-gate	Cradle-to-gate
Life Cycle Assessment Impact method	ReCiPe 2016 [47].	ReCiPe 2016 [47].	IMPACT 2002+ vQ2.28 (Humbert et al. 2012 cited in Khan et al. [41]).	IMPACT 2002+ vQ2.28 (Humbert et al. 2012 cited in Khan et al. [41]).
Allocation	Mass and economic	Mass and economic	Economic	Economic
Environmental impacts (units in parenthesis)				
	Greenhouse gas emissions (kg CO_2_-eq)	Greenhouse gas emissions (kg CO_2_-eq)	Climate change (kg CO_2_-eq)	Change (kg CO_2_-eq)
	Land use (m2a crop eq)	Land use (m2a crop eq)	Land occupation (m2.y)	Land occupation (m2.y)
	Water consumption (L)	Water consumption (L)	Water consumption (L)	Water consumption (L)
	Fossil resource scarcity (kg oil eq)	Fossil resource scarcity (kg oil eq)	Aquatic eutrophication (g PO_4_-eq)	Aquatic eutrophication (g PO_4_-eq)

^1^ Uncooked patty delivered to retail distribution outlets. ^2^ Frozen, ready-to-ship patty.

**Table 2:** Comparisons of the environmental indicators of each PBMA and its respective beef patty comparator data (based on a 4 oz uncooked patty).

Indicator	Unit	Beyond Burger^® 1^	Beef Patty ^1^	Impossible Burger^® 2^	Beef Patty ^2^
GWP	kg CO_2_-eq	0.430	4.260	0.396	3.460
95% CI (lower and upper bound values)		0.418, 0.435	N/A	0.350, 0.452	2.859, 4.238
Water consumption	liter	6.45	219.24	12.07	96.06
95% CI for (lower and upper bound values)		5.93, 7.20	N/A	6.43, 22.97	69.82, 139.91
Monetization	USD_2025_/4 oz patty	0.07	1.01	0.08	0.68
95% CI for monetized values (lower and upper bound values) ^3^		0.07, 0.08	N/A	0.06, 0.10	0.56, 0.80

^1^ Heller and Salim [40], Table 13. ^2^ Khan et al. [41], Table 14. The 95% CIs were provided in the original papers, except for the Beyond Burger^®^ study, where no data were provided. ^3^ The 95% CIs for the monetized values were calculated as described in Supplementary Materials Text S2. N/A: not available.

**Table 3:** Key health-risk-related nutrient content, in grams of nutrient per 100 g of product (based on a 3 oz cooked patty).

Nutrient	Beef Patty ^1^	Beyond Burger^® 2^	Impossible Burger^® 2^
Polyunsaturated fatty acids (PUFAs)	0.516	2.941	3.059
Calcium	0.024	0.015	0.153
Sodium	0.075	0.352	0.325
Dietary fiber	0	1.647	2.635
Trans fatty acids (TFAs)	0.782	0	0
Red meat ^3^	100	0	0

^1^ https://fdc.nal.usda.gov/food-details/171797/nutrients, accessed on 8 August 2025; ^2^ Harnack et al. [49], Tables 4, 5 and 7; ^3^ “Red meat” is not a nutrient, but it is a category used for the HENI calculations since there is a specific DRF for it (as explained in the text), and obviously beef is red meat.

**Table 4:** Dietary risk per nutrient (in µDALY/gram), total dietary risk of each product per gram (in TDR/gram), as well as per patty and its corresponding monetary value (based on a 3 oz cooked patty).

Nutrient	Mean DRF ^1^	Beef Patty	Beyond Burger^®^	Impossible Burger^®^
PUFAs	−0.6	−0.0031	−0.0176	−0.0184
Calcium	−5.1	−0.0012	−0.0008	−0.0078
Sodium	13.9	0.0104	0.0489	0.0451
Fiber other	−0.99	0.0000	−0.0163	−0.0261
Red meat ^2^	0.099	0.0990	0.0000	0.0000
TFAs	4.4	0.0344	0.0000	0.0000
TDR		0.1395	0.0142	−0.0071
TDR/3 oz patty		11.8604	1.2038	−0.6042
Monetary values		1.63	0.16	−0.08
95% CI for monetary values (lower and upper bound values) ^3^		0.82, 2.32	0.08, 0.24	−0.24, 0.05

^1^ Source: Table S3 in the Supplementary Information of Stylianou et al. [48]. ^2^ Specific DRF for red meat associated with the risk of colorectal cancer and Type 2 Diabetes mellitus. ^3^ The 95% CIs for the monetized values were calculated as described in Supplementary Materials Text S2.

**Table 5:** Monetized externalities, market prices, and calculated true costs per product (using USD_2025_ and based on a 3 oz cooked patty).

Indicator	Beyond Burger^®^	Beef Patty	Impossible Burger^®^	Beef Patty
Global warming	0.06	0.63	0.06	0.51
Water consumption	0.01	0.37	0.02	0.16
TDR	0.16	1.63	−0.08	1.63
Summed cost of externalities ^1^	0.24	2.63	0.00	2.30
Market retail price ^2^	1.97	1.16	2.25	1.16
True cost ^3^	2.21	3.80	2.24	3.47
95% CI for true cost (lower and upper bound values) ^4^	2.13, 2.29	N/A	2.10, 2.39	2.71, 4.23

^1^ Sum of the monetized values of global warming, water consumption and TDR of each product. ^2^ Market prices obtained from www.walmart.com, accessed on 10 April 2025. ^3^ Sum of the cost of externalities and market price of each product. N/A: not available since no CIs for beef were provided in the original paper. ^4^ The 95% CIs for the monetized values were calculated as described in Supplementary Materials Text S2; see also Table S8.

**Table 6:** Market price premiums and corresponding difference for cost of externalities of PBMAs with respect to conventional beef (USD_2025_/3 oz cooked patty).

Indicator	Beyond Burger^®^	Impossible Burger^®^
Difference in retail price relative to beef (*Ppbma* − *Pbeef*)	0.81	1.08
Difference in the cost of externalities relative to beef (*Epbma* − *Ebeef*)	−2.39	−2.31
Absolute value of the benefit-to-premium ratio (*R*)	2.96	2.13
95% CI for *R* (lower and upper bound values) ^1^	N/A	1.56, 2.70

^1^ More detailed information on the 95% CIs are provided in Table S9. N/A: not available since no CIs for beef were provided in the original paper. The ratio *R* expressed in absolute value.

**Table 7:** Market price premiums and corresponding difference for cost of externalities of PBMAs with respect to conventional beef assuming a neutral effect of TFAs (Panel A), and of calcium (Panel B) (USD_2025_/3 oz cooked patty).

	Panel A. Neutral Effect of TFAs for Beef		Panel B. Neutral Effect of Calcium	
Indicator	Beyond Burger^®^	Impossible Burger^®^	Beyond Burger^®^	Impossible Burger^®^
Difference in retail price relative to beef (*Ppbma* − *Pbeef*)	0.81	1.08	0.81	1.08
Difference in the cost of externalities relative to beef (*Epbma* − *Ebeef*)	−1.99	−1.91	−2.40	−2.23
Absolute value of the benefit-to-premium ratio (*R*)	2.47	1.76	2.97	2.06
95% CI for *R* (lower and upper bound values) ^1^	N/A	1.29, 2.24	N/A	1.47, 2.65

^1^ More detailed information on the 95% CIs is provided in Table S10 for the case of neutral effect of TFAs and Table S11 for the case of neutral effect of calcium. N/A: not available since no CIs for beef were provided in the original paper. The ratio *R* expressed in absolute value.

**Table 8:** Sensitivity analysis for varying the social cost of carbon, the monetization factor for the DALY, and the difference in retail prices between the PBMAs and their beef comparison.

	Baseline ^1^		Maximum ^2^		Wider spread ^3^		Minimum ^4^	
*Ppbma-Pbeef* ^5^	Beyond Burger^®^	Impossible Burger^®^	Beyond Burger^®^	Impossible Burger^®^	Beyond Burger^®^	Impossible Burger^®^	Beyond Burger^®^	Impossible Burger^®^
+20%	2.47	1.78	2.90	2.04	1.75	1.02	0.97	0.56
+10%	2.69	1.94	3.17	2.22	1.90	1.12	1.06	0.61
**Observed**	**2.96**	**2.13**	**3.48**	**2.44**	**2.10**	**1.23**	**1.17**	**0.68**
−10%	3.29	2.37	3.87	2.71	2.33	1.37	1.30	0.75
−20%	3.70	2.66	4.35	3.05	2.62	1.54	1.46	0.85

^1^ Baseline: Social cost of carbon = USD_2025_ 0.15/kg CO_2_-eq, Monetization factor for µDALY = USD_2025_ 0.137. ^2^ Maximum: Social cost of carbon = USD_2025_ 0.26/kg CO_2_-eq, Monetization factor for µDALY = USD_2025_ 0.137. ^3^ Wider spread: Social cost of carbon = USD_2025_ 0.26/kg CO_2_-eq, Monetization factor for µDALY = USD_2025_ 0.032. ^4^ Minimums: Social cost of carbon = USD_2025_ 0.063/kg CO_2_-eq, Monetization factor for µDALY = USD_2025_ 0.032. ^5^ Difference in retail prices between a PBMA and its beef comparison expressed in plus/minus percentage value with respect to the observed prices (in bold). For Beyond Burger^®^, USD_2025_ = 0.81, and USD_2025_ = 1.08 for Impossible Burger^®^.

## Data Availability

The original contributions presented in the study are included in the article and Supplementary Materials, further inquiries can be directed to the corresponding author.

## Supplementary Materials

The following supporting information can be downloaded at https://www.mdpi.com/article/10.3390/foods15111949/s1, Text S1: Methodology for calculating total dietary risks; Text S2: Methodology for calculating confidence intervals; Text S3: Additional considerations regarding the nutritional differences between beef and PBMAs; Table S1: Calcium, sodium, and dietary fiber content of products in Daily Value (DV) reference values; Table S2: Nutrient content for a 3 oz cooked portion (85 g) by product; Table S3: Normalized nutrient content by product (gram of nutrient/gram of cooked product); Table S4: Dietary risks factors; Table S5: Mean, lower and upper bounds of 95% confidence interval for the DRFs for each nutrient by product; Table S6: Data used for calculating the 95% confidence intervals (B = Baseline, U = Upper bound, L = Lower bound); Table S7: Results from the calculations of the variance and the confidence interval; Table S8: Retail prices and associated baseline (B), 95% Upper (U) and Lower (L) bounds for monetized impacts and true costs of the different products; Table S9: 95% confidence intervals for the *R* ratios associated with each product; Table S10: 95% confidence intervals for the R ratios associated with each product assuming a neutral effect for TRF in the HENI; Table S11: 95% confidence intervals for the R ratios associated with each product assuming a neutral effect for calcium in the HENI; Table S12: Content of key nutrients (% of Daily Values (DV) per 3 oz cooked patty). Excel file “Data_comparing_PBMA_beef_patty.xsls” containing all the data and calculations presented in the tables. Refs. [48,49,51,66,71,72,73,74] are cited in the Supplementary Materials.
