# Supplementary material for: Quantifying the Relationship Between Key Potential Environmental and Nutritional Health Benefits of Plant-Based Meat Alternatives and Their Price Premiums Compared to Beef
Source: Foods. 2026 Jun 1;15(11):1949. doi: 10.3390/foods15111949 (PMC13256792; doi:10.3390/foods15111949)
Supplement: Supplementary file 1 [file foods-15-01949-s001.zip › Supplemental Material.pdf]

Supplemental Material

Text S1: Methodology for calculating total dietary risks

To calculate the total dietary risks for each PBMA, the content of calcium, polyunsaturated fats (PUFA), trans-fatty acids (TFA), calcium, sodium, and fiber were retrieved from Harnack et al. [1]. The data are reported in different tables there. Particularly the data for calcium, sodium, and dietary fiber were reported as % DV which is the percentage of the Daily Value for each nutrient in a serving of the food. The authors also provided the actual DV value for each nutrient in the relevant physical units (e.g. grams, milligrams) (Table S1).

Table S1: Calcium, sodium, and dietary fiber content of products in Daily Value (DV) reference values.

	Daily Value (DV) reference values [1]	Beef Burger [2]	Beyond Burger [3]	Impossible Burger [3]
	quantity	% DV	% DV	% DV
Calcium	1300	2%	1%	10%
Sodium	2300	2%	13%	12%
Dietary fiber	28	0%	5%	8%

Source: Harnack et al. [1]: [1] Table 1, calcium and sodium in mg, dietary fiber in grams; [2] Table 8 % DV for calcium and sodium; [3] Table 5 % DV for calcium and sodium and Table 7 for dietary fiber.

Based on this information the nutrient content for calcium, sodium, and dietary fiber were calculated per 3 oz cooked portion (85 grams). The other nutrients were obtained directly from the different tables in Harnack et al. paper (Table S2). The nutrient content for beef was obtained from the USDA FoodDataCentral Database for broiled ground beef 80% lean/ 20% fat for a 3 oz cooked broiled patty [2]. The nutrients were normalized to one gram of nutrient per gram of product by dividing the amounts by 85 (Table S3).

Table S2: Nutrient content for a 3 oz cooked portion (85 grams) by product.

Nutrient	Unit/3oz	Beef Burger [1]	Beyond Burger [2,3]	Impossible Burger [2,3]
Polyunsaturated fatty acids (PUFA)	g	0.439	2.5[2]	2.6[2]
Calcium	mg	20.4	13[3]	130[3]
Sodium	mg	63.8	299[3]	276[3]
Dietary fiber	g	0	1.4	2.24
Meat	g	85	0	0
Trans fatty acids (TFA) [4]	g	0.67	0	0

Source: [1] Beef, ground, 80% lean meat / 20% fat, patty, cooked, broiled. https://fdc.nal.usda.gov/food-details/171797/nutrients (accessed 8/8/2025). Harnack et al. [1], [2] Table 4; [3] calculated from nutrient data from Harnack et al. (reported in Table S1 above); [4] Harnack et al. [1], Table 7.

Table S3: Normalized nutrient content by product (gram of nutrient/gram of cooked product).

Nutrient	Unit/g	Beef Burger	Beyond burger	Impossible Burger
Polyunsaturated fatty acids (PUFA)	g	0.00516	0.02941	0.03059
Calcium	g	0.00024	0.00015	0.00153
Sodium	g	0.00075	0.00352	0.00325
Dietary fiber	g	0	0.01647	0.02635
Meat	g	1	0	0
Trans fatty acids (TFA)	g	0.00782	0	0
Red meat	g	1	1	1

The dietary risk of each nutrient was calculated by multiplying the nutrient content (Table S2) by its corresponding Dietary Risk Factor (DRF) (Table S4) and then added together to obtain the Total Dietary Risk (TDR) for a product per gram (equation 2 in our paper). Finally, these numbers were converted to a 3 oz patty by multiplying by 85. The mean, lower, and upper bounds for a 95% CI for the different products are presented in Table S5. In this context, negative numbers indicate a benefit (µDALY avoided), and positive numbers indicate a cost (µDALY incurred).

Table S4: Dietary risks factors.

Dietary risk	DRF (µDALYs/g)		
	Mean	Lower	Upper
Polyunsaturated fatty acids (PUFA)	-0.6	-0.26	-0.94
Trans fatty acids (TFA)	4.4	3.3	5.6
Calcium	-5.1	-4	-6.2
Sodium	13.9	11.5	16.1
Fiber other	-0.99	-0.71	-1.3
Red meat	0.099	0.038	0.15

Source: Table S3 in the Supplementary Information of Stylianou et al. [3]

Table S5: Mean, lower and upper bounds of 95% CI for the DRFs for each nutrient by product.

	Conventional beef			Beyond burger			Impossible Burger		
	Mean	Lower	Upper	Mean	Lower	Upper	Mean	Lower	Upper
Polyunsaturated fatty acids (PUFA)	-0.0031	-0.0013	-0.0049	-0.0176	-0.0276	-0.0076	-0.0184	-0.0288	-0.0080
Calcium	-0.0012	-0.0010	-0.0015	-0.0008	-0.0009	-0.0006	-0.0078	-0.0095	-0.0061
Sodium	0.0104	0.0086	0.0121	0.0489	0.0566	0.0405	0.0451	0.0523	0.0373
Fiber other	0.0000	0.0000	0.0000	-0.0163	-0.0214	-0.0117	-0.0261	-0.0343	-0.0187
Red meat	0.0990	0.0380	0.1500	0.0000	0.0000	0.0000	0.0000	0.0000	0.0000
Trans fatty acids (TFA)	0.0344	0.0258	0.0438	0.0000	0.0000	0.0000	0.0000	0.0000	0.0000
Total/g	0.1395	0.0701	0.1996	0.0142	0.0066	0.0205	-0.0071	-0.0202	0.0046
Total/3 oz patty	11.8604	5.9625	16.9620	1.2038	0.5633	1.7425	-0.6042	-1.7184	0.3876
Total/3 oz patty without TFA	8.9344	3.7680	13.2380	N/A	N/A	N/A	N/A	N/A	N/A
Total/3 oz patty without calcium	11.9644	6.0441	17.0885	1.2701	0.6439	1.7945	0.0588	-0.9124	0.9076

N/A: not applicable.

Text S2: Methodology for calculating confidence intervals

To calculate the confidence intervals (CI) for the monetized value of the impacts examined here as well as for the true value of the products, we retrieved the baseline, 95% lower and upper bounds, for GWP and water consumption from the original papers, and for the HENI from our calculations (Table S6, Panel A), which where monetized (Table S6, Panel B). For these calculations we assume that each of the impacts is distributed normally and independently. Based on the lower (L_i) and upper (U_i) bounds of each impact i, we recovered the standard deviation with the following formula:

$$\sigma_i = \frac{U_i - L_i}{2 \times 1.96} = \frac{U_i - L_i}{3.92}.$$

We also sum up the baseline values (μ_i) for each impact i:

$$\mu_S = \sum_i \mu_i .$$

We calculated the variance for each impact i by squaring its standard deviation σ_i and then we summed up the three variances to obtain the global variance and took its square root (Table S7):

$$\mathrm{Var}\ (S) = \sum_i \sigma_i^2 , \sigma_S = \sqrt{\mathrm{Var}\ (S)}.$$

Finally, we obtain the 95% CI (CI) for the sum of the baseline values of impacts:

$$\mathrm{CI}_{95\%} = \mu_S \pm 1.96\, \sigma_S.$$

Results are presented in Table S8.

Table S6: Data used for calculating the 95% CI (B=Baseline, U=Upper bound, L=Lower bound). N/A not available due to lack of data.

	Unit	Beyond Burger®			Beef patty			Impossible Burger®			Beef patty		
		B	L	U	B	L	U	B	L	U	B	L	U
Panel A. Original units													
GWP	kg CO2 eq/4 oz uncooked patty	0.425	0.418	0.435	4.26	N/A	N/A	0.396	0.35	0.452	3.46	2.859	4.238
Water consumption	liter/4 oz uncooked patty	6.45	5.93	7.2	219.24	N/A	N/A	12.07	6.43	22.97	96.06	69.82	139.91
HENI with TFA	µDALY /3 oz patty	1.20	0.56	1.74	11.86	5.96	16.96	-0.60	-1.72	0.39	11.86	5.96	16.96
HENI w/o TFA	µDALY /3 oz patty	N/A	N/A	N/A	8.93	3.77	13.24	N/A	N/A	N/A	8.93	3.77	13.24
HENI w/o calcium	µDALY /3 oz patty	1.27	0.64	1.79	11.96	6.04	17.09	0.06	-0.91	0.91	11.96	6.04	17.09
Panel B. Monetization													
GWP	USD2025/4 oz uncooked patty	0.06	0.06	0.06	0.63	N/A	N/A	0.06	0.05	0.07	0.51	0.43	0.63

Water consumption	USD2025/4 oz uncooked patty	0.01	0.01	0.01	0.37	N/A	N/A	0.02	0.01	0.04	0.16	0.12	0.24
HENI	USD2025/3 oz patty	0.16	0.08	0.24	1.63	N/A	N/A	-0.08	-0.24	0.05	1.63	0.82	2.32
HENI w/o TFA	USD2025/3 oz patty	N/A	N/A	N/A	1.22	N/A	N/A	N/A	N/A	N/A	1.22	0.52	1.81
HENI w/o calcium	USD2025/3 oz patty	0.17	0.09	0.25	1.64	0.83	2.34	0.01	-0.13	0.12	1.64	0.83	2.34

N/A= not applicable

Table S7: Results from the calculations of the variance and the CI. N/A not available due to lack of data.

		Beyond Burger®	Beef patty	Impossible Burger®	Beef patty
GWP	Variance	0.00000042	N/A	0.00001498	0.00273784
Water consumption	Variance	0.00000031	N/A	0.00005206	0.00093483
HENI with TFA	Variance	0.00169889	N/A	0.00541886	0.14782347
HENI w/o TFA	Variance	N/A	N/A	N/A	0.10957172
HENI w/o calcium	Variance	0.00161748	0.14903221	0.00404701	0.14903221
HENI with TFA	95% CI	0.08080375	N/A	0.14517101	0.76288110
HENI w/o TFA	95% CI	N/A	N/A	N/A	0.65957536
HENI w/o calcium	95% CI	0.07884480	N/A	0.12571600	0.76591846

Table S8: Retail prices and associated baseline (B), 95% Upper (U) and Lower (L) bounds for monetized impacts and true costs of the different products (USD_{2025}/3 oz patty). N/A not available due to lack of data.

		Monetized value of impacts			True cost		
	Retail price	B	L	U	B	L	U
Beyond Burger®	1.97	0.24	0.16	0.32	2.21	2.13	2.29
Beef	1.16	2.63	N/A	N/A	3.80	N/A	N/A
Impossible Burger®	2.25	0.00	-0.15	0.14	2.24	2.10	2.39
Beef	1.16	2.30	1.54	3.07	3.47	2.71	4.23

Table S9: 95% CI for the *R* ratios associated with each product using all dietary risks in the HENI.

	Retail price	Monetized value of externalities		
		B	L	U
(1) Beyond Burger®	1.97	0.24	0.16	0.32
(2) Beef	1.16	2.63	N/A	N/A
(3) Impossible Burger®	2.25	0.00	-0.15	0.14
(4) Beef	1.16	2.30	1.54	3.07
Differences	(a)	(b)	(c)	(d)
Beyond Burger® vs. beef (1)-(2)	0.81	-2.39	N/A	N/A
Impossible Burger® vs. beef (3)-(4)	1.08	-2.31	-1.69	-2.92
Ratio *R*		(b)/(a)	(c)/(a)	(d)/(a)
Beyond Burger® vs. beef		2.96	N/A	N/A
Impossible Burger® vs. beef		2.13	1.56	2.70

The first four rows are the same as in the first four columns in Table S8. The numbers and letters in parentheses identify which numbers were used to calculate the differences and ratios for the baseline and the lower (L) and upper (U) 95% CI. N/A not available since the lower and upper bounds were not provided in the original study.

Table S10: 95% CI for the *R* ratios associated with each product assuming a neutral effect for TRF in the HENI.

	Retail price	Monetized value of externalities		
		B	L	U
(1) Beyond Burger®	1.97	0.24	0.16	0.32
(2) Beef	1.16	2.23	N/A	N/A
(3) Impossible Burger®	2.25	0	-0.15	0.14
(4) Beef	1.16	1.90	1.24	2.56
Differences	(a)	(b)	(c)	(d)
Beyond Burger® vs. beef (1)-(2)	0.81	-1.99	N/A	N/A
Impossible Burger® vs. beef (3)-(4)	1.08	-1.91	-1.39	-2.42
Ratio *R*		(b)/(a)	(c)/(a)	(d)/(a)
Beyond Burger® vs. beef		2.47	N/A	N/A
Impossible Burger® vs. beef		1.76	1.29	2.24

The first four rows are the same as in the first four columns in Table S8. The numbers and letters in parentheses identify which numbers were used to calculate the differences and ratios for the baseline and the lower (L) and upper (U) 95% CI. N/A not available since the lower and upper bounds were not provided in the original study.

Table S11: 95% CI for the *R* ratios associated with each product assuming a neutral effect for calcium in the HENI.

	Retail price	Monetized value of externalities		
		B	L	U
(1) Beyond Burger®	1.97	0.25	0.17	0.33
(2) Beef	1.16	2.65	N/A	N/A
(3) Impossible Burger®	2.25	0.09	-0.04	0.21
(4) Beef	1.16	2.32	1.55	3.08
Differences	(a)	(b)	(c)	(d)
Beyond Burger® vs. beef (1)-(2)	0.81	-2.40	N/A	N/A
Impossible Burger® vs. beef (3)-(4)	1.08	-2.23	-1.59	-2.87
Ratio *R*		(b)/(a)	(c)/(a)	(d)/(a)
Beyond Burger® vs. beef		2.97	N/A	N/A
Impossible Burger® vs. beef		2.06	1.47	2.65

The first four rows are the same as in the first four columns in Table S8. The numbers and letters in parentheses identify which numbers were used to calculate the differences and ratios for the baseline and the lower (L) and upper (U) 95% CI. N/A not available since the lower and upper bounds were not provided in the original study.

Text S3: Additional considerations regarding the nutritional differences between beef and PBMAs.

Animal meat is an important source of key nutrients for human nutrition such as iron, zinc, niacin, riboflavin, and vitamins B6 and B12, whose intake could be insufficient in plant-based diets without careful meal planning that includes consuming fortified foods and supplements [1]. A comparison of content of these nutrients for the two PBMAs and the beef analyzed here shows that beef has a higher content of zinc, niacin, and vitamin B12, but Impossible Burger® has a higher content of iron, riboflavin, and Vitamin B6, while Beyond Burger® has no content of riboflavin, Vitamins B6 and B12 (Table S12). Regarding iron, although both PBMAs have higher content than beef, the heme iron of animal-based foods is better absorbed than the non heme iron of plant-based foods [1]. However, an innovation of Impossible Burger® is its content of heme iron from soy leghemoglbin, which potentially has comparable bioavailability to beef, but evidence is still lacking [1]. Furthermore, among those nutrients that are generally higher in beef compared to the PBMAs analyzed here (e.g., zinc, niacin, and B12), none are particularly under consumed among most adults in developed countries [4]. It should be noted however that some sub-groups of the population are at higher risk of underconsumption (such as older adults), but rates of underconsumption remain generally low for these nutrients in particular [5].

Table S12: Content of key nutrients (% of Daily Values (DV) per 3 oz cooked patty).

Nutrient	Conventional beef	Beyond burger	Impossible Burger
Iron	12	13	**17**
Zinc	**48**	5	38
Niacin	**36**	19	25
Riboflavin	11	0	**23**
Vitamin B6	16	0	**18**
Vitamin B12	**99**	0	94

Source: Harnack et al. [1], Table 8 and supplemental Tables 5 and 6. Highest content in bold.

Although the protein quantity of the PBMAs is similar to beef, its quality based on the Digestible Indispensable Amino Acid Score (DIAAS), a widely used metric to assess protein bioavailability [6], is not similar, since meat is an excellent source of protein, with a score of 100, while soy and pea, the base ingredients of these PBMAs, are considered just good sources of protein, with scores of 90 and 82 respectively [7]. However, while protein quality is higher in meat than PBMAs, there is not a real concern over total protein intake among US adults [8], therefore consuming lower-quality protein from PBMAs would not make an appreciable difference in overall quality of protein intake.

References

1. Harnack, L.; Mork, S.; Valluri, S.; Weber, C.; Schmitz, K.; Stevenson, J.; Pettit, J. Nutrient Composition of a Selection of Plant-Based Ground Beef Alternative Products Available in the United States. *J. Acad. Nutr. Diet.* **2021**, *121*, 2401-2408.e12, doi:10.1016/j.jand.2021.05.002.

2. U.S. Department of Agriculture Beef, Ground, 80% Lean Meat / 20% Fat, Patty, Cooked, Broiled Available online: https://fdc.nal.usda.gov/food-details/171797/nutrients (accessed on 8 August 2025).

3. Stylianou, K.S.; Fulgoni, V.L.; Jolliet, O. Small Targeted Dietary Changes Can Yield Substantial Gains for Human Health and the Environment. *Nat. Food* **2021**, *2*, 616–627, doi:10.1038/s43016-021-00343-4.

4. Freedman, M.R.; Fulgoni, V.L.; Lieberman, H.R. Temporal Changes in Micronutrient Intake among United States Adults, NHANES 2003 through 2018: A Cross-Sectional Study. *Am. J. Clin. Nutr.* **2024**, *119*, 1309–1320, doi:10.1016/j.ajcnut.2024.02.007.

5. Wallace, T.C.; Frankenfeld, C.L.; Frei, B.; Shah, A.V.; Yu, C.-R.; Van Klinken, B.J.-W.; Adeleke, M. Multivitamin/Multimineral Supplement Use Is Associated with Increased Micronutrient Intakes and Biomarkers and Decreased Prevalence of Inadequacies and Deficiencies in Middle-Aged and Older Adults in the United States. *J. Nutr. Gerontol. Geriatr.* **2019**, *38*, 307–328, doi:10.1080/21551197.2019.1656135.

6. Berardy, A.J.; Rubín-García, M.; Sabaté, J. A Scoping Review of the Environmental Impacts and Nutrient Composition of Plant-Based Milks. *Adv. Nutr.* **2022**, *13*, 2559–2572, doi:10.1093/advances/nmac098.

7. Katare, B.; Yim, H.; Byrne, A.; Wang, H.H.; Wetzstein, M. Consumer Willingness to Pay for Environmentally Sustainable Meat and a Plant-based Meat Substitute. *Appl. Econ. Perspect. Policy* **2023**, *45*, 145–163, doi:10.1002/aepp.13285.

8. Shan, Z.; Rehm, C.D.; Rogers, G.; Ruan, M.; Wang, D.D.; Hu, F.B.; Mozaffarian, D.; Zhang, F.F.; Bhupathiraju, S.N. Trends in Dietary Carbohydrate, Protein, and Fat Intake and Diet Quality Among US Adults, 1999-2016. *JAMA* **2019**, *322*, 1178, doi:10.1001/jama.2019.13771.
